# Effect of the Interaction between Depression and Sleep Disorders on the Stroke Occurrence: An Analysis Based on National Health and Nutritional Examination Survey

**DOI:** 10.1155/2021/6333618

**Published:** 2021-10-19

**Authors:** Jia Li, Leijun Li, Yan Lv, Yanhai Kang, Mingjin Zhu, Wenfeng Wang

**Affiliations:** ^1^Department of Psychiatry and Psychology, Hainan General Hospital, Haikou, 570311 Hainan, China; ^2^Department of Psychiatry, The Third Affiliated Hospital of Sun Yat-sen University, Guangzhou 510630, China; ^3^Department of Neuorology, Hainan General Hospital, Haikou, 570311 Hainan, China; ^4^Rehabilitation Department, Tongde Hospital of Zhejiang Province, Hangzhou, 310012 Zhejiang, China; ^5^School of Science, Shanghai Institute of Technology, Shanghai 201418, China

## Abstract

Objective. To investigate the effect of the interaction between depression and sleep disorders on the stroke occurrence based on the data from the National Health and Nutritional Examination Survey (NHANES). Methods. Seven cycles of 2-year NHANES data (2005-2018) were analyzed in this study. Univariate analysis was first performed between the stroke and nonstroke patients, and then, multivariate logistic regression models were conducted to analyze the association of depression, sleep disorders, and their interactions with stroke occurrence. Results. A total of 30473 eligible participants were included in this study, including 1138 (3.73%) with stroke and 29335 (96.27%) with nonstroke. Except sex, the differences were all significant between the stroke and nonstroke patients in baseline information (all *P* < 0.001). Depression (odds ratio (OR): 2.494, 95% confidence interval (CI): 2.098-2.964), depression severity (moderate, OR: 2.013, 95% CI: 1.612-2.514; moderately severe, OR: 2.598, 95% CI: 1.930-3.496; severe, OR: 5.588, 95% CI: 3.883-8.043), and sleep disorders (OR: 1.677, 95% CI: 1.472-1.910) were presented to be associated with an increased risk of stroke after correcting all the confounders. The logistic regression analysis showed that there was a synergic, additive interaction between depression and sleep disorders on the stroke occurrence, and the proportion of stroke patients caused by this interaction accounted for 27.1% of all the stroke patients. Conclusion. Depression, depression severity, and sleep disorders are all independently associated with a high risk of stroke. The interaction between depression and sleep disorders can synergistically increase the stroke occurrence.

## 1. Introduction

Stroke is the second most common cause of deaths secondary to ischemic heart disease and a leading cause of disability worldwide [1]. As the population ages, its incidence increases significantly, especially in low- and middle-income countries. According to statistics, there were almost 6.5 million deaths from stroke and 25.7 million stroke survivors in 2013, while 12 million deaths and 70 million survivors would be estimated by 2030 [2, 3]. Despite advances in prevention and treatment in recent years, stroke remains to be the important cause of long-term disability [4]. Stroke survivors are reported to be associated with a high risk of stroke recurrence and adverse events; they were not only at high risk of dementia, but also at high risk of depression, with the prevalence of 29%, which maintained stability up to 10 years following stroke [5–7]. In addition, the prevalence of sleep disorders after stroke was also estimated to be greater than 50% [8].

As a frequent complication of stroke, depression can worsen the course of poststroke neurological disorders, accelerate the patients' physical helplessness, decrease the quality of life, and affect the ability of patients to engage in rehabilitation therapies, consequently leading to an increased risk of death [3]. There is considerable evidence showing that stroke has a complicated bidirectional relationship with depression: stroke can increase the risk of depression occurrence, while depression is confirmed to be a significant risk factor for stroke and stoke mortality [9–12]. Moreover, sleep-disordered breathing and sleep-wake disturbances are also very common in stroke patients [13]. Previous studies indicated that preexisting sleep disorders were associated with an increased risk of stroke and depression and might aggravate after stroke onset, which exerted adverse effects on the outcomes of stroke patients [14–16]. Notably, hypersomnia or insomnia has been a key diagnostic criterion of depression [17]. However, relatively little is known about whether there exists a synergistic effect between sleep disorders and depression and how they interact on the stroke occurrence.

In this study, we attempted to assess the effect of the interaction between depression and sleep disorders on the stroke occurrence based on the data from the National Health and Nutritional Examination Survey (NHANES).

## 2. Materials and Methods

### 2.1. Data Source

The data used in this study were extracted from the NHANES, an ongoing cross-sectional survey representing a noninstitutionalized civilian United States (US) population. NHANES was conducted by the National Center for Health Statistics and Centers for Disease Control and Prevention, aiming at assessing the health status of the US population [18]. The interviews were performed by the study teams which were composed of medical and health technicians, multilingual physicians, and dietary health interviewers, and the information collected was designed to evaluate the incidence of major diseases and risk factors for diseases to promote health and prevent diseases [19].

In this study, seven cycles of 2-year NHANES data (2005-2018) including baseline demographic data and drug use questionnaire information were retrospectively analyzed. Both home interviews and comprehensive physical examinations in a mobile examination center were conducted for all the participants signing the informed consent forms. The participants without depression questionnaire data or sleep disorder data or those lacking other information were excluded. This study did not need to be approved by the Institutional Review Board of Hainan General Hospital because the data used were accessed from the NHANES, a publicly available database.

During the home interview, the demographic data were collected, including age, gender, body mass index (BMI), ethnicity, marital status, educational level, family income, and history of alcohol consumption and smoking. In addition, the presence or absence of sleep disorders, depression, diabetes mellitus, and stroke were also recorded. Sleep disorders were defined according to the item “have you ever told sleep disorders by doctors or professional health workers” from the NHANES. As a short, self-administered questionnaire, the Patient Health Questionnaire 9 (PHQ-9) is widely employed to screen depression in primary care settings [20]. Regarding the severity, depression was classified into five categories based on total PHQ-9 scores, including no depressive symptoms (0-4), mild depressive symptoms (5-9), moderate depressive symptoms (10-14), moderately severe depressive symptoms (15-19), and severe depressive symptoms (20-27) [21]. In this study, depression was defined when the total PHQ-9 scores were equal to or greater than 10 [22].

### 2.2. Statistical Analysis

For the measurement data, a normality test was performed using the Shapiro-Wilk test. The normally distributed data were presented as the mean ± standard deviation ($\bar{x}\pm s$), while abnormally distributed data were as the median and quartile (*M* (*Q*_1_, *Q*_3_)), and *t*-test and Mann–Whitney *U* rank-sum test were conducted, respectively. The enumeration data were compared using the *χ*^2^ or Fisher's exact test, manifesting as cases and the constituent ratio (*n* (%)). The variables with statistical significance in the univariate analysis were enrolled into the multivariate logistic regression model for analyzing the association of depression, sleep disorders, and their interactions with stroke occurrence.

Three indexes including relative excess risk of interaction (RERI), attributable proportion of interaction (API), and synergy index (SI) were used to assess the interaction based on the addictive model. No interactions were shown when 0 was comprised in the confidence interval (CI) of RERI and AP and 1 was involved in the CI of SI.

SAS software (SAS Institute Inc., Cary, NC, USA; version 9.4) was used to manage the data in both univariate and multivariate analyses. The forest plots and interaction schematic diagrams were drawn using R software (R Foundation for Statistical Computing, Vienna, Austria; version 4.2), and pictures of interactive odds ratio (OR) values were drawn using GraphPad Prism 8 software. All the statistical tests were two-sided, and the value of *P* less than 0.05 was statistically significant.

## 3. Results

### 3.1. Baseline Information of Participants

Between 2005 and 2018, there were 44,635 participants in the NHANES database. After excluding 8,411 participants with missing depression questionnaire data, 29 with missing sleep disorder data, and 5,722 with incomplete information, 30,473 participants including 15,043 males and 15,430 females were enrolled into the study, with the median age of 49 years.

Among the included 30,473 participants, 1,138 (3.73%) had stroke and 29,335 (96.27%) did not. The baseline information of the stroke and nonstroke patients was compared in Table 1. It could be observed that the patients with stroke had older age (*P* < 0.001) and higher BMI (*P* < 0.001), as well as higher proportions of smoking (*P* < 0.001), diabetes mellitus (*P* < 0.001), sleep disorders (*P* < 0.001), depression (*P* < 0.001), and depression severity (*P* < 0.001) than those without. Compared with nonstroke patients, stroke patients had lower proportions of alcohol consumption (*P* < 0.001), educational levels (*P* < 0.001), and family income (*P* < 0.001). In addition, significant differences were also shown between the stroke and nonstroke patients in ethnicity (*P* < 0.001) and marital status (*P* < 0.001; Table 1).

### 3.2. Association of Depression and Its Severity with Stroke

**Figure 1:**
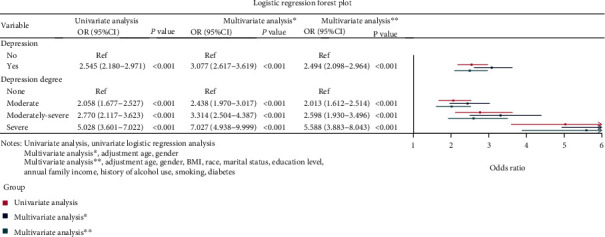
Logistic regression forest plot of the association of depression and its severity with stroke.

The association of depression and its severity with stroke was depicted in Figure 1. As shown, the risk of stroke in patients with depression was 2.545-fold than those without (OR: 2.545, 95% CI: 2.180-2.971, *P* < 0.001). The patients with depression had 3.077-fold of stroke risk than those without (OR: 3.077, 95% CI: 2.617-3.619, *P* < 0.001) after correction of age and sex, and this risk (OR: 2.494, 95% CI: 2.098-2.964, *P* < 0.001) was still present when all the confounders, such as age, sex, BMI, ethnicity, marital status, educational levels, family income, diabetes mellitus, and history of alcohol consumption and smoking, were adjusted.

In terms of depression severity, the patients with moderate (OR: 2.058, 95% CI: 1.677-2.527, *P* < 0.001), moderately severe (OR: 2.770, 95% CI: 2.117-3.623, *P* < 0.001), and severe depressive symptoms (OR: 5.028, 95% CI: 3.601-7.022, *P* < 0.001) all had a markedly increased risk of stroke than those without. When the age and sex were adjusted, the risk of stroke was, respectively, increased by 1.438-fold in patients with moderate depressive symptoms (OR: 2.438, 95% CI: 1.970-3.017, *P* < 0.001), 2.314-fold in patients with moderately severe depressive symptoms (OR: 3.314, 95% CI: 2.504-4.387, *P* < 0.001), and 6.027-fold in patients with severe depressive symptoms (OR: 7.027, 95% CI: 4.938-9.999, *P* < 0.001) compared with those without. After adjusting all the confounders like age, sex, BMI, ethnicity, marital status, educational levels, family income, diabetes mellitus, and history of alcohol consumption and smoking, the risk of stroke was still higher in patients with moderate (OR: 2.013, 95% CI: 1.612-2.514, *P* < 0.001), moderately severe (OR: 2.598, 95% CI: 1.930-3.496, *P* < 0.001), and severe depressive symptoms (OR: 5.588, 95% CI: 3.883-8.043, *P* < 0.001) than those without.

**Figure 2:**
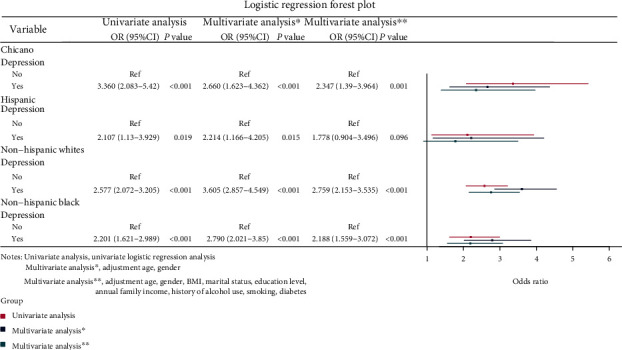
Logistic regression forest plot of the association between depression and stroke according to ethnicities.

Ethnicity-based subgroup analysis of the association between depression and stroke was presented in Figure 2. It could be found that after all the confounders, such as age, sex, BMI, ethnicity, marital status, educational levels, family income, diabetes mellitus, and history of alcohol consumption and smoking, were corrected, the risk of stroke was significantly higher in patients with depression in Chicano (OR: 2.347, 95% CI: 1.390-3.964, *P* = 0.001), non-Hispanic white (OR: 2.759, 95% CI: 2.153-3.535, *P* < 0.001), and non-Hispanic black populations (OR: 2.188, 95% CI: 1.559-3.072, *P* < 0.001) than those without, but not in Hispanic population (OR: 1.778, 95% CI: 0.904-3.496, *P* = 0.096).

### 3.3. Association between Sleep Disorders and Stroke

**Figure 3:**
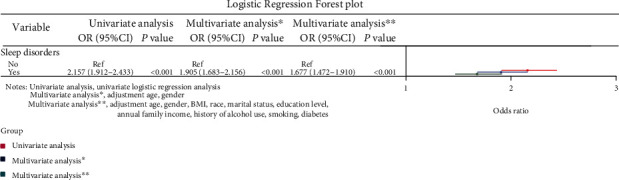
Logistic regression forest plot of the association between sleep disorders and stroke.

Univariate analysis indicated that the risk of stroke in patients with sleep disorders was 2.157-fold higher than those without (OR: 2.157, 95% CI: 1.912-2.433, *P* < 0.001; Figure 3). In multivariate analysis, the patients with sleep disorders were found to have a significantly elevated risk of stroke than those without after adjusting the age and sex (OR: 1.905, 95% CI: 1.683-2.156, *P* < 0.001), and this association still existed even though all the confounders were adjusted (OR: 1.677, 95% CI: 1.472-1.910, *P* < 0.001; Figure 3).

### 3.4. Effect of the Interaction between Depression and Sleep Disorders on Stroke

The interactive items between depression and sleep disorders were established, including no sleep disorders and no depression, no sleep disorders and depression, sleep disorders and no depression, and no sleep disorders and no depression. According to these interactive items, the baseline information of participants was summarized in Table 2.

**Figure 4:**
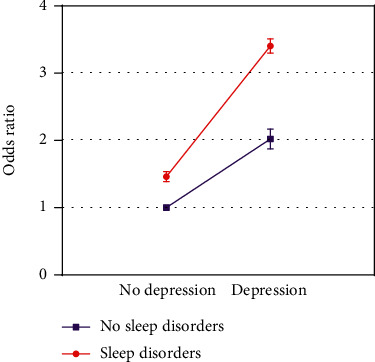
Visualization of odds ratio values and standard deviations in model 3.

**Figure 5:**
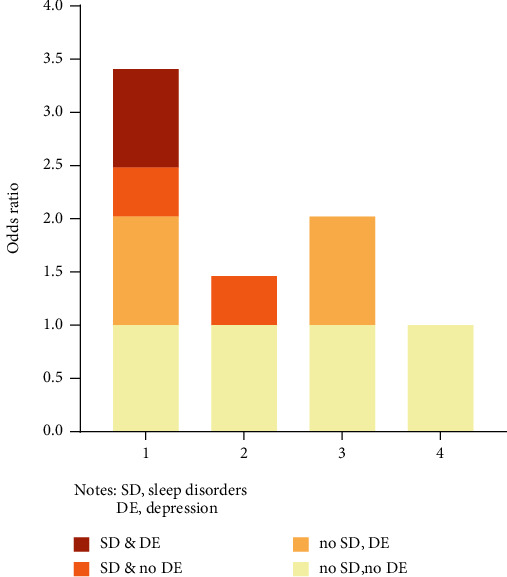
An interaction schematic diagram between depression and sleep disorders after correction of multiple confounders.

The logistic regression analysis revealed that after correcting all the confounders like age, sex, BMI, ethnicity, marital status, educational levels, family income, diabetes mellitus, and history of alcohol consumption and smoking, the CIs of interactive indexes RERI_model2_ (1.131; 95% CI: 0.104-2.158), RERI_model3_ (0.924; 95% CI: 0.078-1.769), API_model2_ (0.263; 95% CI: 0.055-0.471), and API_model3_ (0.271; 95% CI: 0.055-0.487) did not comprise 0, and 1 was not involved in the CIs of SI_model2_ (1.521; 95% CI: 1.019-2.269) and SI_model3_ (1.624; 95% CI: 1.008-2.614), suggesting that there was a synergic, additive interaction between depression and sleep disorders on the stroke occurrence. The API was equal to 0.271 after the variables with significant differences in the univariate analysis were adjusted, which indicated that the proportion of stroke patients caused by the interaction between depression and sleep disorders accounted for 27.1% of all the stroke patients included in this study (Table 3). The OR values and standard deviations in model 3 were visualized in Figure 4, and the interaction schematic diagram between depression and sleep disorders was shown in Figure 5.

## 4. Discussion

In the current study, 30,473 eligible participants were enrolled, containing 1,138 with stroke and 29,335 with nonstroke. We found that depression, depression severity, and sleep disorders were independently correlated with an elevated risk of stroke, and there was a synergic, additive interaction between depression and sleep disorders on the stroke occurrence. The proportion of patients with stroke caused by the interaction between depression and sleep disorders accounted for 27.1% of all the stroke patients.

As a frequent mental disorder, depression can occur repeatedly or for a long time, considerably affecting the capability of individuals to function in their daily life [23]. Amount of evidence has confirmed that depression is closely correlated with stroke occurrence [24–26]. In a meta-analysis, depression was identified to be associated with high risk of ischemic, fatal, and total stroke [27]. Our results showed that patients with depression had 3.077-fold of stroke risk than those without. Mai and Liang also supported that the risk of stroke was higher in patients with moderate, moderately severe, and severe depressive symptoms [28].

Depression may result in stroke through multiple mechanisms. First, depression may affect the risk of stroke through neuroendocrine and inflammation/immunological effects. There is a study showing that depression is positively correlated with interleukin- (IL-) 1, C-reactive protein (CRP), and IL-6 in community and clinical samples [29]. These inflammatory markers have been demonstrated to connect with the occurrence and prognosis of stroke [30]. Second, depression relates to obesity and poor health behaviors, such as smoking, physical inactivity, poor diet, and medication compliance [31, 32], which may raise the risk of stroke. Third, depression is associated with a variety of major comorbidities, including hypertension and diabetes mellitus which are considered to be significant risk factors for stroke [33, 34].

Sleep exerts a significant effect on the human life. Appropriate sleep duration (6-8 h) showed a protective effect on the stroke occurrence, while short or long sleep durations might significantly raise the risk of stroke [28, 35, 36]. Enormous studies have confirmed that sleep disorders are modifiable risk factors for stroke and have an impact on stroke outcome [37–39]. Our results revealed that patients with sleep disorders had 1.677-fold of stroke risk than those without, similar to 1.622 times reported by Mai and Liang [28].

It is well known that sleep disorders are the most dominant symptom in patients with depression and also generally considered the major secondary manifestations of depression. Nevertheless, there is evidence that insomnia is not only a prodromal manifestation of depression but also an independent risk factor for the development and recurrence of depression [15, 40]. There may be a complex bidirectional relationship between sleep disorders and depression, but not a cause-effect relationship [41]. In this study, we analyzed the effect of the interaction between sleep disorders and depression on the stroke occurrence and found that the interaction between sleep disorders and depression could increase the risk of stroke synergistically.

Although amount of evidence has shown that depression and sleep disorders are independent risk factors for stroke, few studies still report the association between their interactions and the risk of stroke. Our study first exhibited a synergic, additive interaction between depression and sleep disorders on the stroke occurrence. Moreover, our results might be more convincing due to the nationally representative nature of NHANES and a large sample size. However, the following limitations should be taken into consideration. First, the participants included in our study might have less severe stroke because noninstitutionalized participants were only involved in the NHANES. Second, some important factors like the stroke severity and stroke types were missing in the NHANES; thus, the stroke severity, a consistent predictor of poststroke depression, was not adjusted in the multivariate analysis. Third, the history of stroke in the NHANES was confirmed through self-reported data. Despite no validation for the self-reporting stroke in the NHANES, the history of stroke from questionnaires had been checked. In several studies, the risk factors for cardiovascular diseases had been identified through the self-reported data from NHANES [42–44].

## 5. Conclusions

Depression, depression severity, and sleep disorders were all independently related to an increased risk of stroke. The combination of depression and sleep disorders might play a synergistic role to increase the occurrence of stroke. In addition, evidence suggested that depression and sleep disorders may affect cardiovascular disease through multiple common pathways [45, 46]. Therefore, depression and sleep disorders may also have common pathways for the impact of stroke. In the future, more prospective studies should be conducted to further verify our results.

## Figures and Tables

**Table 1:** Comparison on the baseline information of the stroke and nonstroke patients.

Variables	Total (*n* = 30,473)	Grouping		*Z*/*χ*^2^/*t*	*P*
		Nonstroke (*n* = 29,335)	Stroke (*n* = 1,138)		
Age (years, *M* (*Q*_1_, *Q*_3_))	49 (34, 64)	48 (34, 63)	68 (58, 77)	31.017	<0.001
Sex, *n* (%)				0.028	0.867
Male	15,043 (49.37)	14,484 (49.37)	559 (49.12)		
Female	15,430 (50.63)	14,851 (50.63)	579 (50.88)		
Body mass index (kg/m^2^, $\bar{x}\pm s$)	29.25 ± 7.00	29.23 ± 7.01	29.97 ± 6.77	-3.460	<0.001
Ethnicity, *n* (%)				97.628	<0.001
Chicanos	4,698 (15.42)	4,599 (15.68)	99 (8.70)		
Hispanics	2,753 (9.03)	2,691 (9.17)	62 (5.45)		
Non-Hispanic whites	13,533 (44.41)	12,950 (44.15)	583 (51.23)		
Non-Hispanic blacks	6,493 (21.31)	6,177 (21.06)	316 (27.77)		
Others	2,996 (9.83)	2,918 (9.95)	78 (6.85)		
Marital status, *n* (%)				382.769	<0.001
Married	15,834 (51.96)	15,289 (52.12)	545 (47.89)		
Widowed	2,368 (7.77)	2,130 (7.26)	238 (20.91)		
Divorced/separated	4,402 (14.45)	4,178 (14.24)	224 (19.68)		
Unmarried	7,869 (25.82)	7,738 (26.38)	131 (11.51)		
Education, *n* (%)				-7.480	<0.001
<High school	7,121 (23.37)	6,791 (23.15)	330 (29.00)		
High school	7,093 (23.28)	6,762 (23.05)	331 (29.09)		
> high school	16,259 (53.36)	15,782 (53.80)	477 (41.92)		
Family income, *n* (%)				164.030	<0.001
<20000$	6,513 (21.37)	6,096 (20.78)	417 (36.64)		
≥20000$	23,960 (78.63)	23,239 (79.22)	721 (63.36)		
History of alcohol consumption, *n* (%)	20,989 (68.88)	20,314 (69.25)	675 (59.31)	50.430	<0.001
History of smoking, *n* (%)	13,922 (45.69)	13,218 (45.06)	704 (61.86)	124.666	<0.001
Diabetes mellitus, *n* (%)	4,042 (13.26)	3,629 (12.37)	413 (36.29)	544.863	<0.001
Sleep disorders, *n* (%)	7,853 (25.77)	7,375 (25.14)	478 (42.00)	162.847	<0.001
Depression, *n* (%)	2,647 (8.69)	2,434 (8.30)	213 (18.72)	149.954	<0.001
Depression severity, *n* (%)				12.497	<0.001
No	27,826 (91.31)	26,901 (91.70)	925 (81.28)		
Moderate	1,649 (5.41)	1,540 (5.25)	109 (9.58)		
Moderately severe	713 (2.34)	651 (2.22)	62 (5.45)		
Severe	285 (0.94)	243 (0.83)	42 (3.69)		

**Table 2:** The baseline information of participants according to interactive items between depression and sleep disorders.

Stroke	Depression		Sleep disorders	OR	
	Yes	No		Depression (yes)	Depression (no)
Yes	150	328	Yes	R11	R10
No	1,392	5,983			
Yes	63	597	No	R01	R00
No	1,042	20,918			

**Table 3:** Logistic regression analysis of the interactive items between depression and sleep disorders.

Sleep disorders	Depression	Model 1			Model 2			Model 3		
		OR	95% CI	*P*	OR	95% CI	*P*	OR	95% CI	*P*
0	0	Ref.			Ref.			Ref.		
0	1	2.118	1.622-2.767	<0.001	2.543	1.928-3.353	<0.001	2.020	1.512-2.699	<0.001
1	0	1.921	1.674-2.205	<0.001	1.628	1.414-1.875	<0.001	1.461	1.260-1.693	<0.001
1	1	3.776	3.132-4.552	<0.001	4.302	3.536-5.235	<0.001	3.405	2.766-4.190	<0.001
RERI (95% CI)		0.736 (-0.124-1.597)			1.131 (0.104-2.158)			0.924 (0.078-1.769)		
API (95% CI)		0.195 (-0.011-0.401)			0.263 (0.055-0.471)			0.271 (0.055-0.487)		
SI (95% CI)		1.361 (0.932-1.989)			1.521 (1.019-2.269)			1.624 (1.008-2.614)		

Notes: Model 1: univariate logistic regression analysis; Model 2: multivariate logistic regression analysis after correcting the age and sex; Model 3: multivariate logistic regression analysis after correcting the age, sex, body mass index, ethnicity, marital status, educational levels, family income, diabetes mellitus, and history of drinking alcohol and smoking.

## Data Availability

The data utilized to support the findings are available from the corresponding authors upon request.
